# Some Experiments Made with a View to Ascertain the Duration of the Infectious Power of Variolous Matter

**Published:** 1786

**Authors:** Thomas Houlston

**Affiliations:** Physician to the Liverpool Infirmary


II. *Some Experiments made with a View to aſcertain the Duration of the infectious Power of variolous Matter. By* Thomas Houlſton, *M. D. Phyſician to the Liverpool Infirmary.*

I HAD always concluded, that the matter of the ſmall pox preſerved for a long time the power of communicating the diſeaſe, and I thought I could pretty confidently aſcribe as the cauſe of a young gentleman's being infected, his ſleeping in a room in which another had paffed through the difeafe fome years before; particular caution having always been employed till then to guard him againft the attack of it. In order to afcertain how far thefe opinions might be confidered as well founded, I have fince made fome trials with matter which I had preferved by me many years. I took it from two children of the late Prince Andrew Poniatowfki, brother to the King of Poland, in whofe inoculation I was employed in 1768, at Teplin, a country feat near Vienna. It was taken, fome upon raw filk, and fome upon a cotton thread, and had remained in my poffeffion, in a bottle flightly corked, above thirteen years, when I employed it in 1781. A part of the filk was carefully applied to an incifion made in the arm of a boy two years old. It produced a degree of inflammation, from whence infection might moft naturally have been expected to enfue; but it did not: and, on thefe appearances gradually going off, I inoculated him again, a month afterwards, with frefh matter, and he had the difeafe regularly.

The year following I introduced a fmall portion both of the thread and filk, moiftened with the variolous matter in 1768, into incifions made made in each arm of a girl four years of age. Every neceſſary caution was obſerved, but no effect followed, though the operation was repeated in the ſame manner a ſecond time; and the parents objected to my again inoculating the child, which I meant to have done with freſh matter. She did not take the diſeaſe till about a year afterwards, when ſhe was attacked with it naturally, and got through it eaſily.

As particular attention was paid to theſe two caſes, and as the matter proved unequal to communicate the ſmall pox, although the ſubjects were very capable of receiving the infection, I deſiſted from any farther trial; and though the event turned out contrary to my expectation, yet it may perhaps be judged worth while to record the reſult of experiments, made with a view to aſcertain whether or not variolous matter, after ſuch a number of years, is ſtill poſſeſſed of ſufficient activity to reproduce the diſeaſe. For ſome years poſſibly it may *; but

* I obſerve that the author of a late Work, ſtiled RUSSIA, ſpeaking of the Kamtſchadales, relates (vol. iii. p. 159.) that "they formerly innoculated their children for the ſmall pox, "by ſcratching the face with a fiſh bone dipped in variolous "matter. As this diſtemper made no appearance for a num- "ber but thefe facts feem to demonftrate, that its power of communicating infection does not continue for fo long a period as fourteen or fifteen years.

*Liverpool,*

Oct. 19, 1785.

---

III. *Cafe of a Retention of Urine removed by Electricity. Communicated in a Letter to Dr.* Simmons, *by* Samuel Snowden, *M. D. Phyfician at Stroud, in Gloucefterfhire.*

I TAKE the liberty to fend you the following cafe, to be inferted, if you pleafe, in the London Medical Journal. It relates to a retention of urine removed by electricity; which being but flightly mentioned by authors, may, perhaps, merit attention. The patient,

" ber of years, they neglected this falutary practice, when

" in 1758 it was unluckily brought there by a foldier, *who*

" *had been long cured of it* *. This fcourge of the human

" race then raged in fo dreadful and fatal a manner, that it

" fwept away two thirds of the nation."

* In the 6th vol. of this Journal, p. 209, the reader will find the refult of Dr. Haygarth's inquiries with refpect to the time at which variolous patients ceafe to be infectious. —Editor.

